# Tissue-resident memory T cells reshape *in situ* immune surveillance at luminal barriers and during early cancer interception

**DOI:** 10.3389/fimmu.2026.1877664

**Published:** 2026-07-15

**Authors:** Yimin Qin, Senyang Guo, Wei Chen, Xuan Ji, Jianhua Liu, Hongmei Zheng

**Affiliations:** Breast Cancer Center, Hubei Cancer Hospital, Tongji Medical College, Huazhong University of Science and Technology, National Key Clinical Specialty Construction Discipline, Hubei Provincial Clinical Research Center for Breast Cancer, Wuhan Clinical Research Center for Breast Cancer, Wuhan, Hubei, China

**Keywords:** luminal barrier, mammary duct, mucosal immunity, multi-omics, spatial biology, tissue-resident memory T cells

## Abstract

Tissue-resident memory T (TRM) cells provide a conceptual framework for understanding how barrier tissues sense, restrict and amplify immune responses at the earliest sites of danger. Rather than viewing memory primarily through the delayed recruitment of recirculating cells, the TRM perspective places immune memory within defined tissue niches, where cellular position, antigen experience, egress restraint, metabolic adaptation and local cellular interactions collectively determine the quality of *in situ* surveillance. Here we outline the conceptual evolution and definitional boundaries of TRM cells, distinguish complementary CD4^+^ and CD8^+^ TRM programmes across luminal barriers, and place particular emphasis on TRM cells in tumour surveillance, immunotherapy response, precancerous niches and mammary duct interception. We also integrate recent human reproductive tract phenotyping data to link TRM states with clinically relevant tissue contexts. We argue that future TRM studies should move beyond CD69 or CD103-based annotation and instead build evidence chains that integrate multi-omics, spatial biology, TCR clonality and functional validation. The mammary duct is not a classical mucosal organ, but it may become a critical setting in which to test how far TRM-based principles of *in situ* immunity can be extended to cancer prevention and local immune intervention.

## 1 How TRM cells reshape *in situ* immune surveillance

The significance of TRM cells is rooted in the time pressure faced by barrier tissues. The respiratory tract, intestine, reproductive tract and urinary tract are all exposed, in different ways, to the external environment. Pathogen seeding, epithelial injury, local cytokine release and immune-cell redistribution can occur within hours. Classical memory T-cell models emphasize the size of the memory pool, the speed of reactivation and the efficiency of recruitment. Those principles remain important, but they cannot fully explain early protection that begins before large numbers of circulating cells have entered the tissue. Skin and mucosal infection models, quantitative tissue surveys and local rechallenge experiments collectively show that pre-positioned memory cells can alter outcomes before pathogens expand or abnormal epithelial cells spread (1–8).

TRM cells shift the question from how many memory cells can be mobilized to whether the appropriate cells are already located at the site where danger first appears. This shift turns *in situ* immune surveillance into a set of testable biological processes: whether cells reside within epithelium, lamina propria, luminal interfaces or tumour boundaries; whether they carry antigen experience and rapid effector potential; whether they are supported by tissue-retention signals and metabolic niches; and whether they interact locally with epithelial cells, dendritic cells, macrophages, B cells or stromal cells. Reviews of TRM biology and human tissue atlases have made clear that tissue location is not a passive background condition; it is part of memory function itself (9–24).

This view also changes the starting point for studying local disease. In infection, TRM cells explain why protective responses can begin before recruitment. In chronic inflammation, they reveal how long-lived memory-like cells may shift from protection to inflammatory amplification. In cancer, they move attention upstream from T-cell infiltration in established tumours to resident-like T-cell networks in premalignant epithelium, lesion borders, post-treatment residual disease and tumour-distant tissues. Studies in breast cancer, melanoma, lung cancer and several other solid tumours show that tissue position, antigen specificity and clonal relationships often explain clinical outcome better than T-cell abundance alone (25–41).

The mucosal immunity cycle further strengthens this tissue-based view. Local antigen capture, priming in draining lymph nodes or mucosa-associated inductive sites, acquisition of organ-homing imprints, return of lymphocytes to the entry tissue, and establishment of TRM cells, tissue-resident memory B cells and local antibody responses are best understood as linked stages of a continuous process (42). In this process, the match between route of entry, inductive site and effector site directly shapes whether local immune memory is deposited and maintained.

## 2 From recirculation to residence: conceptual evolution and definitional boundaries

The TRM concept is not a minor addition to conventional memory T-cell classification. Early memory T-cell biology was organized around central memory T cells and effector memory T cells: the former preferentially recirculate through lymphoid organs, whereas the latter more readily enter peripheral tissues. This framework remains fundamental to recall immunity and vaccine biology (4, 43). Studies of non-lymphoid tissues gradually revealed, however, that some antigen-experienced T cells do not merely pause in inflamed organs. They remain locally after infection or inflammation resolves. These observations first emerged from skin and local viral infection models and were later extended to the lung, intestine, brain, liver, reproductive tissues and human multi-tissue samples, establishing tissue residence as a major form of immune memory (1–3, 5–7, 44–52).

The field then faced a more demanding problem: how should TRM cells be defined? CD69, CD103, CD49a, CXCR6, KLF2-S1PR1 downregulation, local IL-7, IL-15 or TGF-beta signalling, lipid uptake and tissue-specific metabolic adaptation can all contribute to the establishment or maintenance of residence, but no single marker is equivalent to TRM identity across all tissues, diseases and species (20–24, 38, 39). This limitation is particularly clear in human samples and tumours: CD103-negative cells may behave as residents; CD69 upregulation may reflect recent activation; and CD39 or PD-1 may indicate tumour reactivity, chronic stimulation or functional remodelling depending on context.

Recent deep-profiling studies suggest that tissue residence is better understood as a modular state than as a single transcriptional signature. Spatial retention, restrained egress, local restimulation, metabolic adaptation, antigen reactivity and tissue interaction together form the evidentiary basis for TRM annotation (37–40). A more robust definition therefore treats TRM cells as tissue-anchored, antigen-experienced memory cells. They frequently express residence-associated molecules and suppress egress programmes, but their spatial position, local function, clonal history and interactions with the tissue niche are more decisive.

This tighter definition is especially important in cancer. Resident-like CD8 T cells in tumours may be bona fide tumour-reactive TRM cells, bystander cells or exhausted derivatives shaped by persistent TCR stimulation. Distinguishing protective residence from bystander infiltration or exhaustion requires antigen testing, TCR clonal analysis, spatial localization, functional assays and transcriptomic or chromatin-state information (35–40). Definitional discipline does not weaken the TRM concept; it makes it more useful for cross-organ, cross-disease and translational research.

The same definitional discipline is needed when separating CD4^+^ and CD8^+^ TRM programmes. CD8^+^ TRM cells are often emphasized because they provide rapid cytotoxicity, IFN-gamma release and direct control of infected or transformed epithelial cells. Their differentiation and maintenance are commonly linked to TGF-beta-dependent CD103 induction, CD69-associated restraint of egress, local antigen encounter, lipid-metabolic adaptation and survival within epithelial or parenchymal niches (7, 20–23). CD4^+^ TRM cells, however, are not simply accessory populations. In mucosal tissues, CD4^+^ TRM cells can differentiate into Th1-, Th17-, Treg-like or tissue-helper states, persist through local antigen re-encounter and chemokine-supported retention, and localize to airway, intestinal, vaginal, cervical and urinary mucosal compartments where they coordinate macrophage and dendritic-cell networks, provide help to B cells and CD8^+^ T cells, regulate epithelial repair and maintain barrier homeostasis (11, 47, 48). Thus, CD4^+^ and CD8^+^ TRM cells should be interpreted as complementary modules of local memory rather than as interchangeable residents.

## 3 TRM cells at luminal barriers: route of entry, tissue niche and functional consequence

**Figure 1:**
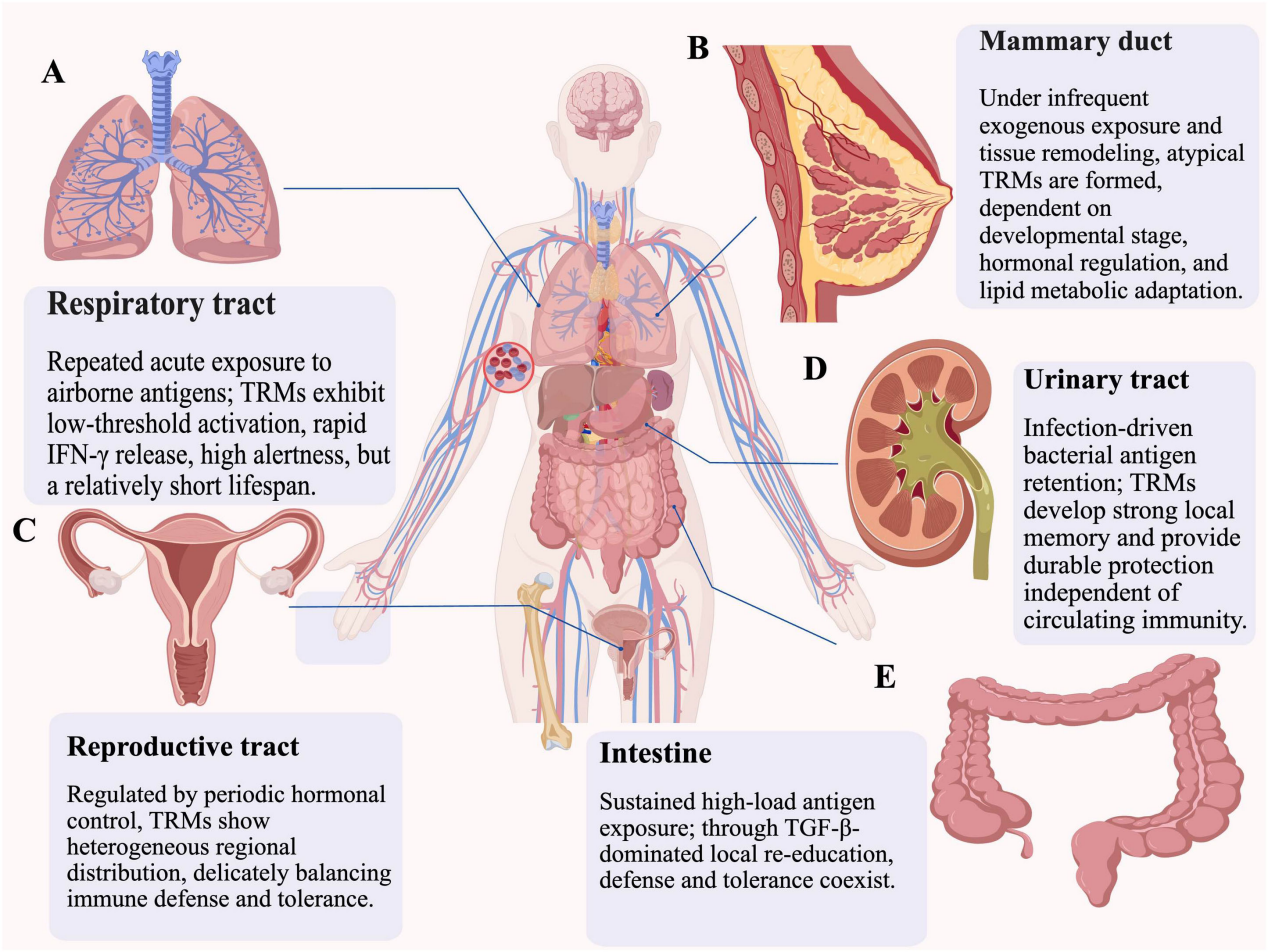
Region-specific immune features and regulatory cues of TRM cells across major luminal barrier tissues. This schematic summarizes how tissue-resident memory T (TRM) cells are shaped by distinct anatomical, environmental and physiological contexts across five luminal barrier sites. **(A)** Respiratory tract: repeated acute exposure to airborne antigens favours rapidly responsive TRM cells with low activation thresholds, prompt IFN-γ release and strong local alert capacity, although these cells may show limited long-term persistence. **(B)** Mammary duct: under relatively infrequent exogenous antigen exposure but recurrent developmental, hormonal and tissue-remodelling cues, atypical TRM-like states may emerge and depend on developmental stage, hormonal regulation and lipid-metabolic adaptation. **(C)** Reproductive tract: cyclic hormonal control and regional anatomical specialization shape heterogeneous TRM distributions, balancing antiviral and antitumour defence with reproductive tolerance. **(D)** Urinary tract: infection-driven antigen retention and epithelial injury–repair cycles support local memory formation, enabling rapid protection during recurrent bacterial challenge with partial independence from circulating immunity. **(E)** Intestine: sustained high-load antigen exposure, microbiota-derived signals and TGF-β-dominated local re-education promote TRM programmes in which immune defence and tolerance coexist. Created with BioGDP.com (92).

Luminal barrier tissues differ from other TRM-containing tissues because immune surveillance is organized along epithelial-lined interfaces that communicate with the external environment through inhalation, ingestion, genital exposure, urinary infection or ductal secretion. Skin studies have been essential for defining fixed immune surveillance in a keratinized stratified barrier, whereas liver TRM cells exemplify residence in a blood-filtering, metabolically specialized and tolerogenic organ rather than a classical luminal surface (1, 2, 19, 20, 22, 51). By contrast, the respiratory tract, intestine, female reproductive tract and urinary tract are shaped by route-specific antigen entry, local inductive pathways, mucus or epithelial turnover, microbiota or recurrent infection, and, in selected sites, pronounced hormonal or developmental remodelling (42, 45–48). These differences explain why luminal barriers do not simply share a single TRM programme. Respiratory tissues highlight repeated acute exposure, the intestine reflects sustained high antigen load, the female reproductive tract is strongly influenced by hormonal and tissue-stage regulation, and the urinary tract illustrates local memory driven by recurrent infection (45–48). Across these barriers, CD8^+^ TRM cells are often positioned as rapid effector and cytotoxic sentinels, whereas CD4^+^ TRM cells more often organize local cytokine tone, epithelial-barrier regulation, myeloid-cell recruitment and immune homeostasis (11, 47, 48). A schematic overview of these region-specific immune contexts is provided in **Figure 1**.

Respiratory TRM cells most clearly illustrate the advantage of speed. The nasal mucosa, airways and lung parenchyma are major entry sites for aerosolized pathogens, and local TRM cells can rapidly release IFN-gamma, TNF and chemokines, placing epithelial cells, dendritic cells and innate immune populations into an alert state. Lung TRM cells are often less durable than their skin or intestinal counterparts, probably reflecting the limited tolerance of lung tissue for sustained inflammation. Intranasal, inhaled and material-enhanced vaccines further show that route of delivery determines which cells first acquire antigen and influences whether TRM cells, resident memory B cells, local antibodies and trained-like innate components are deposited at the portal of entry (45, 50, 53–73). CD8^+^ respiratory TRM cells are therefore most often discussed as rapid antiviral effectors, whereas CD4^+^ airway TRM cells add a regulatory and helper layer: after influenza infection, VLA-1-dependent tissue-memory CD4^+^ T cells persist in the airways, and T-cell help can also shape trained-like innate programmes in the lung (62, 71).

Intestinal TRM cells face a different challenge: stimulation is not scarce, but excessive. Food antigens, commensals, microbial metabolites and intermittent pathogens create a dense antigenic landscape. Local antigen re-encounter, retinoic-acid-associated homing, epithelial turnover, microbiota-derived metabolites and lamina propria niches all shape intestinal residence. These inputs allow TRM cells to maintain selective vigilance during homeostasis, but barrier disruption, dysbiosis or chronic inflammation can reprogramme them into IFN-gamma- or IL-17-linked pathological circuits (46, 52, 69–74). CD8^+^ intestinal TRM cells contribute to epithelial surveillance, cytotoxic recall and tumour-associated resident-like programmes, whereas CD4^+^ TRM cells have disproportionate roles in barrier calibration by coordinating Th17-related responses, regulatory programmes, epithelial repair and crosstalk with antigen-presenting cells (11, 46, 52, 74–80). Broader mucosal and tissue-resident literature reinforces this logic: human mucosal TRM studies highlight disease-specific heterogeneity, memory-cell migration experiments show that tissue entry and retention can diverge, liver-resident CD8 T cells demonstrate front-line residence outside classical mucosa, and intestinal premalignancy and colorectal cancer studies link resident-like programmes to tumour vulnerability and prognosis (75–80). Together, these findings indicate that protective meaning in the intestine must be interpreted through antigen specificity, CD4/CD8 subset composition, epigenetic state, spatial distribution and clonal relationships.

Female reproductive tract TRM cells deserve stronger integration into barrier immunology because some of the most influential early evidence for tissue residence came from genital HSV and HIV studies. The HSV prime-and-pull model showed that local chemokines can recruit antigen-experienced T cells into vaginal tissue and establish protective memory. Vaginal CD4 TRM-cell maintenance depends on a local macrophage chemokine network, indicating that CD4^+^ TRM cells can function as locally retained organizers of antiviral barrier immunity rather than only as helpers for circulating responses (81, 82). Human cervicovaginal studies show that memory T cells in different anatomical compartments of the reproductive tract acquire distinct differentiation programmes and tissue-adapted features (47, 83, 84). Recent human clinical phenotyping further grounds these concepts in translational practice: in women attending a colposcopy clinic, ectocervical samples contained higher immune-cell and T-cell abundance than endocervical cytobrushes, CD4^+^ and CD8^+^ TRM cells were substantially more frequent in the ectocervix, CD4^+^ TRM cells showed Th17- and regulatory-associated marker patterns, and cervical dysplasia or ectropion was associated with altered mucosal immune-cell profiles including lower CD4^+^ TRM abundance (85). HSV-2-specific female reproductive tract TRM cells recognize diverse viral antigens, cervical resident memory T cells can serve as a cellular HIV reservoir, and human herpesvirus studies further support a role for tissue-resident T cells in local antiviral surveillance (86–88). Cervical cancer studies further indicate that CD103-positive tumour-infiltrating T cells can be tumour-reactive and associated with prognostic benefit (89).

Urinary tract TRM studies are fewer in number, but they provide distinctive evidence for local memory. In recurrent urinary tract infection models, bladder and urinary mucosal TRM cells rapidly restrict bacterial burden after rechallenge even when the contribution of recirculating T cells is limited (48). TRM cells therefore do not belong only to classical high-commensal mucosa; they may represent a broader memory strategy in tissues exposed to cycles of epithelial injury, repair and reinfection (48). Studies in urinary tract cancer and inflammation further suggest clinically relevant links among CD103-positive tumour-infiltrating T cells, bladder mucosal TRM cells and local inflammatory states (90, 91). In this setting, CD8^+^ TRM cells are likely to contribute to rapid cytotoxic and inflammatory recall, whereas CD4^+^ TRM cells may have outsized roles in epithelial-barrier regulation, cytokine coordination, myeloid-cell crosstalk and local immune homeostasis, although the mechanisms that maintain these cells in humans require more spatial and functional evidence (48, 91). These tissue-specific TRM niches and functional priorities are summarized in **Table 1**, and the CD4^+^ versus CD8^+^ TRM comparison across luminal barriers is presented in **Table 2**.

**Table 1:** TRM niches, functional priorities and evidence base across luminal barriers.

Barrier tissue	Dominant niches	Induction and maintenance cues	Main functional emphasis	Key constraints	References
Respiratory tract	Nasal mucosa, airways and lung parenchyma	Local antigen deposition, airway APCs, local restimulation and vaccine route	Rapid alarm, antiviral restriction, CD8^+^ effector recall and CD4^+^ helper coordination	Low tolerance of lung tissue for inflammation; comparatively limited TRM durability	(45, 50, 53–73)
Intestine	Intraepithelial compartment, lamina propria and gut-associated inductive sites	Local antigen re-encounter, retinoic-acid homing, microbiota and metabolites	Selective vigilance under high antigen load, with CD4^+^ barrier calibration and CD8^+^ epithelial surveillance	Barrier disruption and chronic inflammation can reshape function	(46, 52, 69–80)
Female reproductive tract	Vagina, cervix and endometrium	HSV/HIV exposure, local chemokine networks, hormonal and tissue stage	Antiviral protection, pathogen persistence, CD4^+^/CD8^+^ TRM specialization and tumour reactivity	Strong anatomical stratification and time dependence	(47, 81–89)
Urinary tract	Bladder epithelium, lamina propria and urinary mucosa	Recurrent infection, epithelial injury and repair	Rapid local protection during bacterial rechallenge and CD4^+^-skewed barrier regulation	Long-term maintenance and human evidence remain limited	(48, 90, 91)
Mammary duct	Ductal epithelium, periductal stroma and premalignant lesion borders	Developmental and hormonal programmes, involutional remodelling, low-level inflammation and local delivery	Potential surveillance of premalignant lesions, resident-helper niche regulation and early interception	Requires spatial, clonal and functional evidence	(93–113)

**Table 2:** Comparative features of CD4^+^ and CD8^+^ TRM cells across luminal barrier tissues.

Tissue context	CD4^+^ TRM features	CD8^+^ TRM features	Functional cooperation	References
Respiratory tract	Airway CD4^+^ memory cells can persist locally, provide cytokine help and support innate or myeloid coordination during respiratory recall.	CD8^+^ TRM cells provide rapid IFN-gamma/TNF release, local alarm and antiviral restriction at nasal, airway and lung-parenchymal sites.	CD4^+^ help can support APC and innate programmes, whereas CD8^+^ TRM cells rapidly reduce pathogen burden.	(45, 50, 53, 62, 71–73)
Intestine	CD4^+^ TRM cells help calibrate tolerance, Th17/regulatory balance, epithelial repair and APC crosstalk under high antigen load.	CD8^+^ TRM cells mediate epithelial surveillance, cytotoxic recall and resident-like tumour-associated responses.	CD4^+^ TRM cells tune inflammatory set-points, while CD8^+^ TRM cells provide lesion- or pathogen-directed effector control.	(11, 46, 52, 69–80)
Female reproductive tract	CD4^+^ TRM cells are sustained by local macrophage chemokine networks and show Th17- or regulatory-associated phenotypes in human cervix.	CD8^+^ TRM cells contribute to HSV-specific surveillance, compartmentalized cervicovaginal memory and tumour-reactive epithelial infiltration.	CD4^+^ TRM-derived cytokine and chemokine circuits help sustain local CD8^+^ recall while balancing antiviral defence with reproductive tolerance.	(47, 81–89)
Urinary tract	CD4^+^ TRM cells are implicated in barrier regulation, epithelial injury-repair responses, myeloid crosstalk and local immune homeostasis.	CD8^+^ TRM cells contribute to rapid inflammatory or cytotoxic recall during recurrent infection and may be represented among CD103+ tumour-infiltrating cells.	CD4^+^ TRM cells organize tissue repair and immune tone, while CD8^+^ TRM cells provide faster effector restriction during rechallenge.	(48, 90, 91)
Mammary duct and early cancer	CD4^+^ resident-helper states remain insufficiently defined but may influence APC licensing, B-cell help, TLS formation and inflammatory restraint.	CD8^+^ TRM-like cells are linked to parity/lactation-associated protection and antitumour resident-memory programmes in breast cancer.	CD4^+^ TRM or resident-helper circuits may condition the niche that enables protective CD8^+^ TRM-like immunity during early interception.	(25–34, 93–113, 126–130)

## 4 TRM cells in cancer: spatial surveillance, antigen reactivity and therapy response

In cancer, the value of TRM biology is not to rename tumour-infiltrating T cells as resident-like cells. Its value is to explain why location, clonal relationships and functional state can alter lesion fate. Solid-tumour studies have shown that CD103-positive or resident-memory-like CD8 T-cell programmes often correlate with favourable outcome. Such associations become most informative when interpreted alongside cancer immunoediting, immune contexture, the tumour immune microenvironment and tertiary lymphoid structures. Outcome is determined not simply by whether T cells are present, but by whether they are located within tumour nests, epithelial borders, stromal regions, tertiary lymphoid structures (TLS)-adjacent areas or tumour-distant tissues, and by how they interact with antigen-presenting cells and stromal architecture (25–34, 114–130).

Although much of the cancer literature has centred on CD8^+^ TRM cells because of their direct cytotoxicity and relationship to exhaustion, CD4^+^ resident-memory and resident-helper states should not be neglected. CD4^+^ TRM cells may regulate antigen-presenting-cell licensing, chemokine production, B-cell and TLS organization, regulatory tone and tissue repair, thereby shaping whether CD8^+^ TRM cells remain protective or enter dysfunctional trajectories (11, 33, 82, 126–130).

Two interpretive traps should be avoided. First, bystander CD8 T cells are abundant in human tumours and can carry activated or resident-like phenotypes without recognizing tumour antigen (114). Second, genuinely tumour-reactive T cells may evolve along trajectories of persistent antigen stimulation and exhaustion, creating partial phenotypic and transcriptional overlap between TRM and TEX states (35–40). CD39/CD103 co-expression, PD-1-linked functional states, clonal replacement after therapy, relationships to stem-like CD8 T cells, antigen specificity and TCR clonal expansion should therefore be considered together when annotating tumour TRM cells (131–137).

TRM cells also have mechanistic value because they can amplify local immunity. Tissue-resident CD8 TRM cells can activate dendritic cells, induce chemokines and promote antigen spreading, thereby converting local recognition into a broader antitumour response (33). In breast cancer, this view converges with long-standing work on tumour-infiltrating lymphocytes, CD8 infiltration, neoadjuvant chemotherapy response and the search for the missing determinants of immunotherapy benefit. Together, these studies suggest that the breast is not an immunological exception; it is a tissue in which spatial and state-resolved interpretation has been underdeveloped (138–144).

Precancerous niche studies move the discussion even further upstream. Whether a lesion persists may be shaped before overt cancer is established, through interactions among stroma, immune cells and abnormal epithelium (41). TRM research in cancer should therefore extend beyond TILs in established tumours to resident immune networks in premalignant epithelium, post-treatment residual lesions, tissues at risk of recurrence and tumour-distant sites.

## 5 Mammary duct TRM cells: from tissue remodelling to early cancer interception

The mammary duct offers a luminal immune setting distinct from classical mucosa. Most breast cancers arise within the ductal-lobular system, and the ductal epithelium and periductal stroma form the early spatial environment of tumour initiation. At the same time, the duct is physically accessible, making local delivery, local sampling and local immune modulation technically conceivable. The mammary duct, however, is not equivalent to airway or intestinal mucosa. It is not continuously exposed to high exogenous antigen load; instead, its immune environment is strongly shaped by ovarian cycling, pregnancy, lactation, involution, tissue repair and age-associated remodelling (93–107).

This distinctive biology raises a more interesting question: can resident-like T cells in the mammary duct be imprinted by developmental and tissue-remodelling events? Parity and lactation induce CD8 T cells with TRM-like features in normal breast tissue and are linked to long-term T-cell-mediated protection against breast cancer (96). This observation moves the field from asking whether breast tumours contain TRM-like cells to asking whether normal or premalignant ducts can acquire protective resident immune imprints. If so, the mammary duct may become a key tissue in which reproductive history, local immune memory and early cancer interception intersect.

The mammary gland is not a static background. Pregnancy-associated breast cancer, postpartum involution, collagen remodelling, COX-2-associated inflammation, alveologenesis, ductal development, pregnancy-associated epigenetic memory and DCIS progression all alter the local epithelial-stromal-immune niche (97–107). Mammary duct TRM research should therefore treat tissue stage as a core variable rather than viewing the breast as a generic tumour site. Whether ductal epithelium at different physiological stages provides distinct retention signals, lipid-metabolic resources or opportunities for antigen exposure to TRM-like cells should be a priority for future work.

From an intervention perspective, *in situ* vaccination, STING activation, radiotherapy-STING combinations, phototherapy combined with immune checkpoint blockade and intraductal drug delivery provide a toolbox for shaping local immunity (108–113). Intraductal chemotherapy studies suggest that local ductal delivery can increase local drug exposure and reduce systemic toxicity (112). At the same time, analyses of immune-checkpoint-inhibitor toxicity remind us that any attempt to amplify immunity *in situ* must weigh tissue cost as carefully as efficacy (113). For the mammary duct, the goal should not be non-specific inflammation, but the controlled establishment of antigen-relevant, spatially precise and longitudinally monitorable resident immune imprints.

A rigorous research path for mammary duct TRM cells should include at least five evidence layers. First, spatial evidence should determine whether resident-like T cells are located in normal ducts, DCIS, invasive fronts or periductal stroma. Second, clonal evidence should test whether their TCRs are linked to premalignant or tumour lesions. Third, functional evidence should demonstrate antigen-associated cytotoxicity, cytokine release or antigen spreading. Fourth, temporal evidence should track pregnancy, lactation, involution, treatment and pre-recurrence stages. Fifth, safety evidence should determine whether local immune intervention induces chronic inflammation, fibrosis or damage to normal mammary function.

## 6 Multi-omics and spatial biology: from marker-based annotation to evidence-chain reasoning

One reason TRM reviews can become repetitive is that many studies still enter the field through CD69 or CD103. The more informative direction is to move from phenotypic naming to evidence-chain annotation, because residence-associated markers alone cannot resolve lineage, antigen specificity, tissue positioning or functional state (20–24, 37–40, 145–150). Multi-omics and spatial biology now allow investigators to ask where the cells are, whether they share clones, whether they are antigen-reactive, what transcriptional or chromatin state they occupy, and whether they retain functional recall capacity (37–40, 145–150).

This evidence chain can be organized into five layers. The phenotypic layer asks whether cells express residence-associated molecules. The spatial layer asks whether the cells occupy disease-relevant niches. The clonal layer asks whether their TCRs connect them to local lesions, adjacent tissue or distant sites. The functional layer asks whether they can rapidly recall, kill, release cytokines or promote antigen spreading. The integrated-state layer distinguishes protective TRM cells from bystanders and exhaustion-derived states. The corresponding evidence-chain framework is summarized in **Table 3**.

**Table 3:** Evidence chain for annotating TRM cells.

Evidence layer	Core question	Recommended approaches	Common overinterpretation	References
Phenotype	Do the cells express residence-associated molecules?	Flow cytometry, multiplex immunofluorescence and single-cell transcriptomics	Equating CD69 or CD103 expression with TRM identity	(20–24, 38, 145, 146)
Spatial evidence	Do the cells occupy disease-relevant niches?	Spatial transcriptomics, multiplex imaging and imaging mass cytometry	Ignoring differences among intraepithelial, boundary-zone, stromal and distal tissue localization	(15, 39, 147–150)
Clonal evidence	Are the cells clonally linked to local lesions or related tissues?	Single-cell TCR sequencing and longitudinal TCR tracking	Interpreting local enrichment as proof of antigen specificity	(27, 37, 131–137)
Functional evidence	Do the cells retain rapid recall and local amplification capacity?	Restimulation, cytotoxicity, cytokine and antigen-spreading assays	Inferring function from static transcriptional states alone	(2, 3, 33)
Integrated state	Are the cells protective TRM cells, bystanders or exhausted derivatives?	Integrated RNA/ATAC/protein/TCR modelling with antigen testing	Collapsing TRM, TEX and bystander cells into one category	(35–40, 145–150)

This methodological shift is particularly important for the mammary duct. A duct-associated TRM-like cluster identified by single-cell RNA sequencing generates a hypothesis, not a conclusion. Ductal TRM cells become biologically meaningful only when spatial imaging places them within duct-associated niches, TCR sequencing links them to premalignant or tumour lesions, functional assays show antigen-related responses, and longitudinal samples connect them with lesion persistence, recurrence or therapeutic response.

## 7 Outlook

TRM cells are reshaping the study of *in situ* immune surveillance at luminal barriers because they change the starting point of the question. The key issue is not only whether more immune cells can be recruited, but whether tissues already contain memory cells that are correctly positioned, appropriately tuned and rapidly callable before danger expands. Respiratory, intestinal, reproductive and urinary tract studies show that TRM cells follow different organ logics related to speed, tolerance, hormonal regulation and recurrent infection.

For cancer research, TRM cells are important because they connect spatial position, antigen specificity, clonal history and therapy response. TILs in established tumours are only part of the story; resident immunity in precancerous niches, epithelial borders, ductal microenvironments and post-treatment residual lesions may determine earlier whether a lesion is eliminated, held dormant or allowed to progress. The mammary duct deserves attention for this reason. It is neither a classical mucosal organ nor an immunological blank space, but a specialized luminal immune site shaped by development, hormones, tissue remodelling and tumour initiation.

The next task is not to label more cells as TRM, but to build evidence chains that reviewers and readers can evaluate: spatial resolution, clonal tracking, functional validation and longitudinal sampling. Along this path, TRM biology may mature from a successful concept in infection immunology into a broader language for understanding tissue disease, early cancer development and local immune intervention.
